# Smartphone-Based Telemonitoring for Better Oral Health With Toothbrushes: 6-Month Randomized Controlled Trial

**DOI:** 10.2196/65128

**Published:** 2025-02-10

**Authors:** Jaeyeon Kim, Yiseul Choi, Yoolbin Song, Wonse Park

**Affiliations:** 1 Department of Advanced General Dentistry Yonsei University College of Dentistry Seoul Republic of Korea; 2 Institute for Innovation in Digital Healthcare Yonsei University Seoul Republic of Korea; 3 Human Identification Research Institute and Oral Science Research Center Yonsei University College of Dentistry Seoul Republic of Korea

**Keywords:** clinical studies, clinical trials, oral hygiene, plaque, plaque biofilms, halitosis, microbiome

## Abstract

**Background:**

A toothbrush device that telemonitors toothbrushing is a technologically advanced solution providing personalized feedback on toothbrushing habits and oral hygiene. These devices integrate smartphone apps to enhance oral health compliance through dental professional feedback.

**Objective:**

This 6-month prospective randomized controlled trial aimed to compare the clinical effectiveness, defined as improved oral hygiene measured by plaque reduction and halitosis control, of an interactive telemonitoring toothbrush (ITT), an oscillating-rotating power toothbrush (ORT), and a manual toothbrush (MT).

**Methods:**

Participants were recruited offline from the Department of Advanced General Dentistry at Yonsei University Dental Hospital, South Korea. A total of 150 participants were randomly assigned to 3 groups (50 participants each): (1) an ITT connected to a smartphone app providing real-time feedback and weekly dental professional reviews, (2) an ORT with smartphone-based guidance requiring participants to send weekly brushing records via screenshots, and (3) an MT with a brushing diary for review. Data collection occurred in clinical settings. Primary outcomes included plaque reduction measured using the Simple Hygiene Score (SHS), while secondary outcomes included plaque reduction measured using the Turesky modification of the Quigley-Hein plaque index (QHI), reductions in halitosis, and changes in oral microbiota. All outcomes were assessed at baseline and 1 month, 3 months, and 6 months.

**Results:**

A total of 150 participants completed the study. Over 6 months, the SHS increased in the MT group (mean 3.16, SD 4.86 to mean 5.66, SD 5.20) but significantly decreased in the ITT group (mean 3.47, SD 5.50 to mean 2.27, SD 3.82; *P*=.004). Similarly, QHI decreased more in the ITT group (mean 1.79, SD 0.72 to mean 0.85, SD 0.63) than in the ORT (*P*<.001) and MT (*P*<.001) groups. Regarding microbiota, there were no significant differences in high-risk periodontal microbiota or the ratio of caries-risk to anticaries microbiota between the ITT and ORT groups. However, in the MT group, the ratio of caries-risk microbiota was significantly higher at the 3-month (*P*<.001) and 6-month (*P*=.005) recalls than at baseline and at the 3-month (*P*=.048) and 6-month (*P*=.03) recalls than at the 1-month recall. Poststudy questionnaires indicated that 45 of 50 ITT participants (92%) and 37 of 50 ORT participants (76%) reported improved brushing ability. The most effective feature in the ITT group was brushing training, while participants in the ORT group cited the brushing guide as most useful (*P*<.001). Satisfaction scores were higher in the ORT group (mean 7.90, SD 1.21) than in the ITT group (mean 7.15, SD 1.66; *P*=.004). The number of brushing events decreased significantly in the ORT group (*P*=.02), while brushing duration increased in the MT group (*P*=.01).

**Conclusions:**

ITTs enable better oral hygiene management than MTs through dental professional feedback. However, further studies are needed to optimize feedback intervals and improve long-term adherence.

**Trial Registration:**

Clinical Research Information Service (CRIS), Republic of Korea, KCT0009094; https://cris.nih.go.kr/cris/search/detailSearch.do?seq=26110&search_page=L

## Introduction

Toothbrushing plays an important role in plaque removal and maintenance of optimal oral health [1]. It can also prevent oral diseases, particularly caries, gingivitis, and periodontitis [2,3]. Moreover, a prior study reported that regular toothbrushing, flossing, and dental visits are essential to prevent dental problems and maintain good oral hygiene [4].

With technological advances, the oral care industry has introduced smart toothbrushes. A smart toothbrush is a technologically advanced toothbrush that uses sensors, connectivity, and data analysis to provide personalized feedback on an individual’s toothbrushing habits and oral hygiene. These toothbrushes have various features, such as a pressure sensor to prevent overbrushing, a timer to ensure that the tooth surfaces in the mouth are brushed for an appropriate amount of time, and a Bluetooth connection that sends data to a mobile app for analysis. Additionally, some smart toothbrushes can be used by professionals as telemonitoring devices for oral care advice.

Software that offers real-time visual feedback on an individual's brushing movements is being developed to improve brushing techniques. The real-time feedback provided by the toothbrush connected to the mobile app allows users to enhance their toothbrushing technique to improve oral hygiene and reduce the risk of dental problems, such as plaque and gingivitis [5]. In addition, these telemonitoring toothbrushes can help users maintain the right habits by monitoring their toothbrushing habits and providing notifications to signal the recommended brushing time [6]. However, relatively few studies have compared the efficacy of telemonitoring and manual toothbrushes; thus, more research on the impact of telemonitoring toothbrushes on oral health is needed [7-9].

We hypothesized that, among smart toothbrushes, toothbrushes with a telemonitoring function might improve users’ oral hygiene compared with other toothbrushes. Therefore, this study aimed to report the results of a 6-month prospective randomized controlled trial to compare the clinical effectiveness of using an interactive telemonitoring toothbrush (ITT) or an oscillating-rotating electric toothbrush (ORT) among smart toothbrushes as well as a manual toothbrush (MT). Furthermore, we compared the overall oral hygiene results regarding plaque, halitosis, dental caries, and periodontal microbiota between the 3 groups using the 3 different types of toothbrushes. In addition, participants’ toothbrushing habits, awareness of telemonitoring toothbrushes, and compliance were also investigated.

## Methods

### Study Design

This study was a parallel-group, randomized, controlled, single-blind clinical trial and was conducted in the Department of Advanced General Dentistry between January 2021 and May 2022. In this trial, the data analysts were blinded to the group assignments to ensure unbiased statistical analysis. This study was registered with the Clinical Research Information Service and is reported following the CONSORT-EHEALTH (Consolidated Standards of Reporting Trials of Electronic and Mobile Health Applications and Online Telehealth) checklist (Multimedia Appendix 1) [10].

### Participants

Participants were recruited via offline recruitment strategies. The study recruited participants through notices posted at Yonsei University Dental Hospital and within Yonsei University. Interested individuals underwent a screening process, including a review of medical records and an oral examination, to confirm eligibility based on the study criteria. The inclusion criteria were as follows: patients (1) with good general health and who were aged >19 years; (2) who had 24 or more teeth, including implants or bridges; and (3) who used a smartphone. The exclusion criteria were as follows: patients (1) with moderate to severe periodontitis [11]; (2) with orthodontic devices in the oral cavity; (3) with removable dentures; (4) with salivary gland–related diseases; (5) with halitosis caused by systemic diseases (eg, digestive system–related diseases, liver-related diseases, kidney-related diseases, medication-related osteonecrosis of the jaw); (6) who had received head and neck radiation therapy; (7) who were pregnant or lactating; (8) who lacked communication skills (including disabled individuals); and (9) whose work was related to dentistry or medicine

The sample size was 150 participants, divided into 3 groups of 50 each. The sample size was calculated using G-Power 3.1.9.2, with an effect size of 0.3, significance level of .05, and statistical power of 85%, considering a dropout rate of 15% [12]. The study participants were randomly divided into 3 groups (ORT, ITT, and MT) using block randomization (block size: 5) via software (Excel, Microsoft Corp) before the start of the study. Participants identified as eligible were randomized using a computerized randomization tool by 2 independent researchers who were not involved in the rest of the study. Before participating in the study, participants who met the inclusion criteria completed a self-reported questionnaire.

### Procedures and Study Test Products

**Figure 1:**
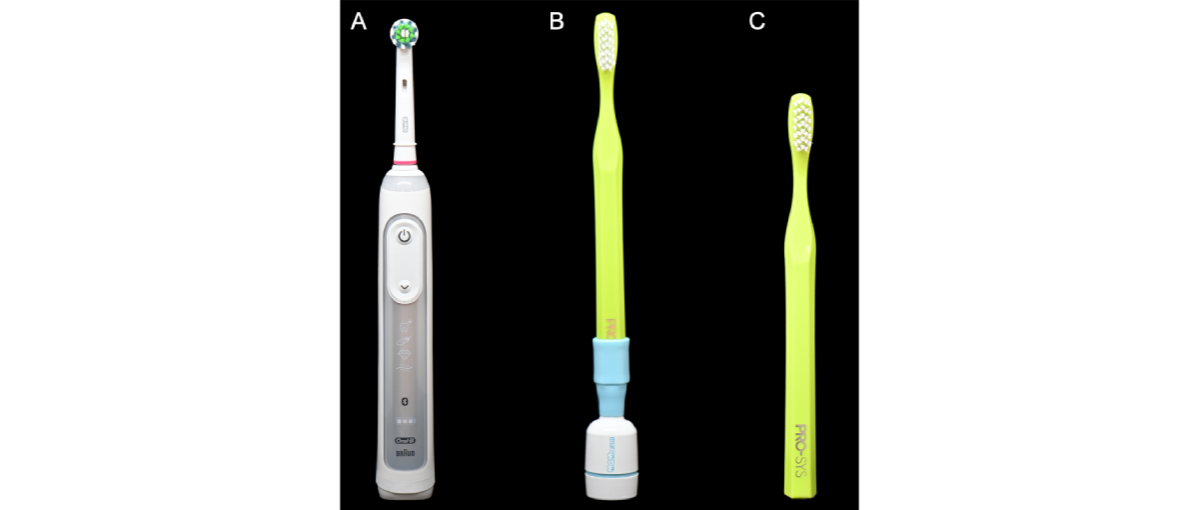
Toothbrush types used in the study: (A) Oral-B Genius 8000 (Procter & Gamble), (B) Mombrush (XiuSolution), (C) manual toothbrush (PRO-SYS Sensitive Toothbrush).

In this study, an MT and 2 types of smart toothbrushes were used: (1) ITT and (2) ORT (Figure 1).

The ITT (Mombrush; XiuSolution) was chosen as the primary intervention due to its advanced telemonitoring capabilities. This toothbrush connects to the Mombrush ProCare smartphone app via Bluetooth, allowing for real-time tracking of brushing behavior, including frequency, duration, and technique.

After installing the Mombrush ProCare app on the participants’ smartphones, they brushed according to the video guide on how to brush with the rolling method (Multimedia Appendix 2). Participants in the ITT group were also registered in the Mombrush advisor application, which allowed the researcher to check the records of continuous brushing habits, regular brushing habits, brushing balance, and brushing areas in real time. These brushing records were stored on a cloud server and reviewed weekly by dental professionals, who provided personalized feedback on regular brushing habits, brushing balance, and brushing zones.

The ORT with a CrossAction brush head (Oral-B Genius 8000; Procter & Gamble), which was included as a comparator, features smartphone-guided brushing without telemonitoring capabilities. It represents an advanced electric toothbrush widely used in practice, enabling a direct comparison with the telemonitoring approach.

Selected participants were instructed to install the Oral-B app and connect the electric toothbrush to their smartphone via Bluetooth (Multimedia Appendix 3). Participants brushed in Clean mode using the coaching function provided by the app. Since this app was an on-device app, the researcher could not check the brushing records in real time, so the researcher received a screenshot of the brushing records once a week and provided feedback.

The American Dental Association reference MT (PRO-SYS Sensitive Toothbrush; Benco Dental) was selected for the control group and serves as the standard brushing method. This allowed for the evaluation of the added benefits provided by digital and telemonitoring interventions.

The MT group recorded the number of brushing strokes in a brushing diary after receiving brushing instruction. Participants in the MT group sent their brushing diaries to the researcher once a week. The diaries were reviewed, and a professional provided feedback.

All participants were instructed to brush their teeth with the products distributed at least twice daily during the study period and to abandon other oral hygiene aids, such as dental floss, interdental brushes, and mouthwashes. All groups were provided with standard sodium fluoride toothpaste (1450 ppm NaF) for use with their assigned toothbrush.

### Clinical Measurement Outcomes

#### Primary Outcome: Simple Hygiene Score (SHS)

To evaluate dental plaque, the following 5 white-light and fluorescent images were captured using the quantitative light-induced fluorescence system (Qraycam; AIOBIO): (1) frontal photograph showing the labial surfaces of the anterior teeth, (2) right and (3) left lateral photographs showing the buccal surfaces of the posterior teeth, (4) maxillary dentition photograph showing the palatal and occlusal surfaces of the maxillary dentition, (5) mandibular dentition photograph showing lingual and occlusal surfaces of the mandibular dentition. The fluorescent plaque index scoring for the fluorescent images of the quantitative light-induced fluorescence system was performed automatically using the Simple Hygiene Score (SHS) with the analysis program Q-ray (version 1.38; Inspektor Research Systems BV). The SHS scores the plaque level from 0 to 5 based on the area of ​​red fluorescent plaque attachment, with a larger plaque attachment area giving a higher score [13].

#### Secondary Outcomes

##### Plaque Reduction

The Turesky modification of the Quigley-Hein plaque index (QHI) was used to measure the presence of plaque [14,15]. The crown and cervical surfaces of the maxillary right first molar, left central incisor, left first premolar, mandibular left first molar, right central incisor, and right first premolar were stained with the disclosing solution (1% neutral red). Plaques were evaluated at 2 sites (buccal and lingual) per tooth, and each site was scored on a scale of 0 to 5 (0=no plaque, 1=slight staining at the cervical margin, 2=plaque band up to 1 mm at the cervical margin, 3=plaque band wider than 1 mm but covering less than one-third of the crown of the tooth, 4=band covering at least one-third but less than two-thirds of the crown of the tooth, and 5=band covering more than two-thirds of the crown of the tooth).

##### Volatile Sulfur Compounds

To measure halitosis, H_2_S, and CH_3_SH concentrations were measured using the Twin Breasor II (iSenLab Inc) with a nanotech semiconductor sensor made by a plasma ion beam. The participants held a straw in their mouth, breathed through their nose for 50 seconds, and exhaled through their mouth for 10 seconds. Exhaled breath was collected by an automatic suction method using a straw, and the sampling gas volume was approximately 10 mL. Analysis was performed 150 seconds after exhalation, and the measurement units were ng/10 mL and ppb.

##### Collection of Saliva Samples

To examine caries-related and periodontal bacteria in the oral cavity, a T-SWAB TRANSPORT UTM (Noble Biosciences) was used to swab the gingiva, cervical region, and tooth area of the right maxillary and mandibular molars for more than 30 seconds. Afterward, the cotton swabs were stored in a collection container containing a preservative solution and frozen at –80 °C before DNA extraction. Bacterial genomic DNA was extracted using the MagNA Pure 96 DNA and Viral NA Small Volume Kit (Roche Diagnostics) according to the manufacturer's instructions. DNA concentration was determined fluorometrically on the Qubit 3.0 Fluorometer (Thermo Fisher Scientific) using the Qubit dsDNA HS Assay Kit. Real-time polymerase chain reaction (PCR) was performed using the PowerCheck Periodontitis Pathogens Multiplex Real-time PCR kit (KogeneBiotech) and PowerCheck Dental Caries Pathogens Multiplex Real-time PCR kit (KogeneBiotech). In this study, *Aggregatibacter actinomycetemcomitans*, *Porphyromonas gingivalis*, *Tannerella forsythia*, and *Treponema denticola* were classified as high-risk periodontal microbiota. *Streptococcus mutans*, *Streptococcus obrinus*, *Actinomyces gerencseriae*, *Scardovia wiggsiae*, *Veillonella parvula*, and *Candida albicans* were classified as caries-risk microbiota, whereas *Streptococcus sanguinis* was classified as anticaries microbiota [16].

The clinical evaluations at baseline and follow-ups were completed by dental professionals in the same dedicated location within the Department of Advanced General Dentistry at Yonsei University Dental Hospital. To ensure consistency and minimize interexaminer variability, the same examiner performed all measurements and assessments for each participant throughout the study. After clinical evaluation at baseline, scaling was performed for all participants, and toothbrushes assigned in advance were distributed. After 1 month, 3 months, and 6 months from the beginning of the study, the participants underwent follow-up clinical evaluations in the same manner as at baseline. At the last visit, the participants completed a self-reported, paper-based questionnaire consisting of 8 questions on current oral care habits and awareness regarding telemonitoring toothbrushes. The dental professional abstracted the responses from the paper-based questionnaires and inputted the data into a database for subsequent analysis.

### Statistical Analysis

All statistical analyses were performed using SPSS (version 25; IBM Corp). Data were tested for normality using the Kolmogorov Smirnov test. One-way ANOVA and chi-square tests were used to analyze the demographics and questionnaire responses of the study participants. Independent-sample *t* tests were used to compare changes in brushing habits by group. SHS, QHI, and volatile sulfur compounds were analyzed using repeated measures ANOVA with Bonferroni multiple comparison tests. Changes in microbiota within groups were analyzed using a paired-samples *t* test. All statistical analyses were 2-tailed, and the statistical significance level was set at *P*<.05.

### Ethical Considerations

The Institutional Review Board (IRB) of the Dental Hospital at Yonsei University reviewed and approved the study protocol prior to the trial in accordance with the ethical standards laid down in the 1964 Declaration of Helsinki and its later amendments (IRB number: 2-2020-0032). All participants completed a written consent form and were given information regarding the study. All data were anonymized to ensure participant confidentiality.

## Results

### Participant Characteristics

**Figure 2:**
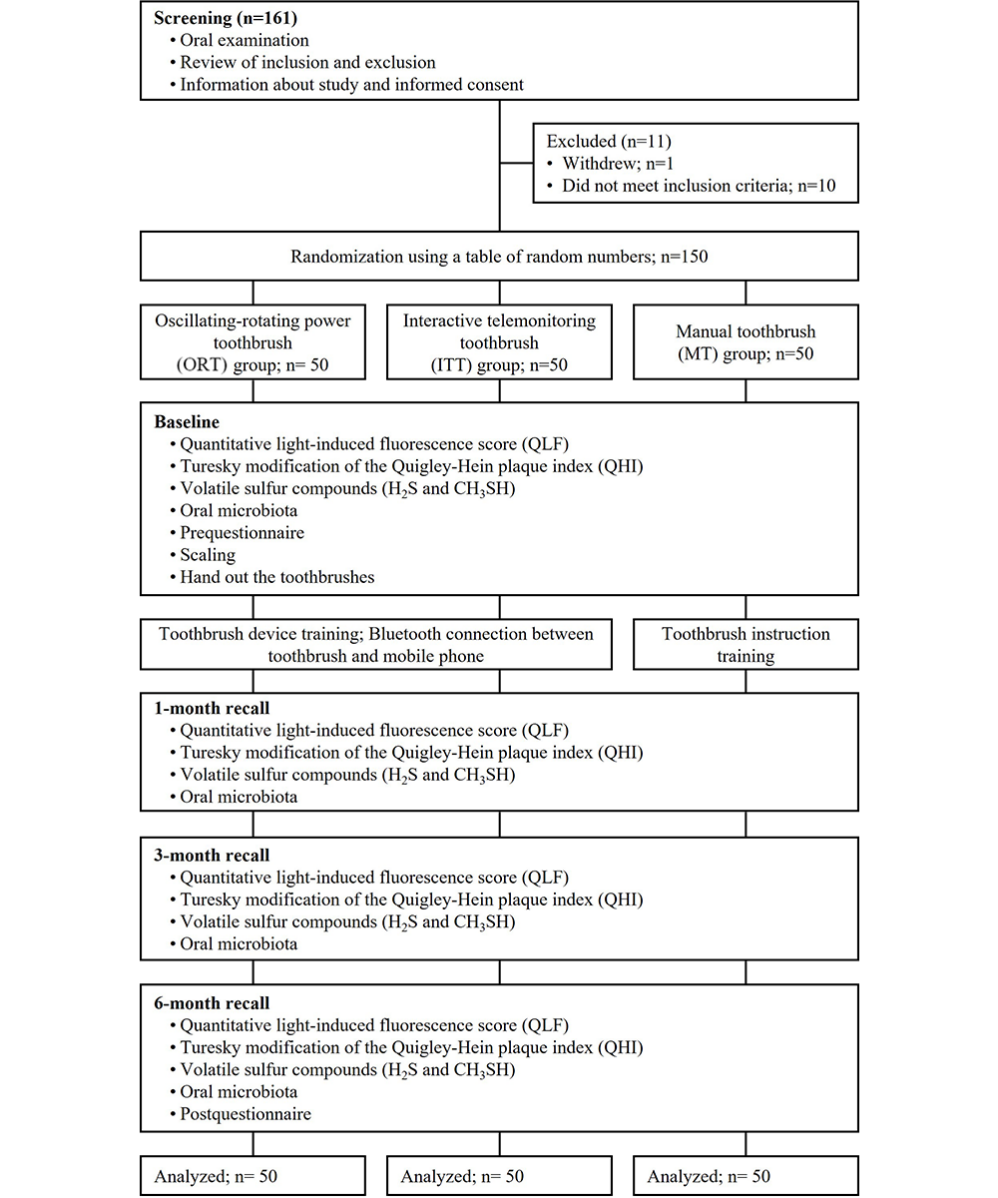
CONSORT-EHEALTH (Consolidated Standards of Reporting Trials of Electronic and Mobile Health Applications and Online Telehealth) flow diagram.

A total of 161 participants completed the screening; 11 of the screened participants did not meet the inclusion criteria, resulting in 150 participants who completed the study (Figure 2). The general characteristics of the participants are presented in Table 1. The average age of the participants was 31.90 (SD 7.34) years, and 61 (61/150, 40.7%) men and 89 (89/150, 59.3%) women were included. Regarding their academic qualifications, a bachelor’s degree was the most common academic qualification in all groups. There were no statistically significant differences in general characteristics among the 3 groups. Similarly, there were no statistically significant differences between the groups in the self-reported questionnaire on current oral care habits and awareness of telemonitoring toothbrushes at the beginning of the study.

**Table 1:** General characteristics and responses to the prequestionnaire on oral care habits and participants’ awareness of telemonitoring toothbrushes.

Characteristics		ORT^a^ (n=50)	ITT^b^ (n=50)	MT^c^ (n=50)	*P* value^d^	
Age (years), mean (SD)		29.10 (4.72)	30.96 (8.39)	32.66 (8.13)	.053	
**Sex, n (%)**						.67
	Male	23 (46)	19 (38)	19 (38)		
	Female	27 (54)	31 (62)	31 (62)		
**Educational level, n (%)**						.96
	High school graduate	10 (20)	10 (20)	9 (18)		
	Associate’s degree	5 (10)	4 (8)	5 (10)		
	Bachelor’s degree	21 (42)	26 (52)	23 (46)		
	Master’s degree or higher	14 (28)	10 (20)	13 (26)		
Number of toothbrushing events, mean (SD)		2.53 (0.62)	2.72 (0.61)	2.70 (0.61)	.28	
Length of each toothbrushing event (minutes), mean (SD)		2.84 (0.75)	3.10 (0.81)	3.12 (0.82)	.20	
**Have you ever received training on oral hygiene management?, n (%)**						.29
	Yes	24 (48)	32 (64)	28 (56)		
	No	26 (52)	18 (36)	22 (44)		
**How long do you think you need training to have a good brushing habit?, n (%)**						.54
	1 year or more	4 (8)	2 (4)	0		
	6 months	3 (6)	3 (6)	2 (4)		
	3 months	6 (12)	8 (16)	9 (18)		
	1 month	13 (26)	15 (30)	20 (40)		
	1 week	24 (48)	22 (44)	19 (38)		
**Do you think that good brushing habits help to maintain healthy teeth?, n (%)**						.42
	Strongly agree	28 (56)	32 (64)	35 (70)		
	Agree	20 (40)	14 (28)	14 (28)		
	Neutral	2 (4)	4 (8)	1 (2)		
	Disagree	0	0	0		
	Strongly disagree	0	0	0		
**Have you ever heard of a telemonitoring toothbrush?, n (%)**						.15
	I know well	1 (2)	1 (2)	1 (2)		
	I know a little	3 (6)	5 (10)	5 (10)		
	I don't know	27 (54)	14 (28)	24 (48)		
	I have never heard	19 (38)	30 (60)	20 (40)		
**Are you interested in trying out a telemonitoring toothbrush?, n (%)**						.87
	Strongly agree	27 (54)	22 (44)	21 (42)		
	Agree	16 (32)	20 (40)	18 (36)		
	Neutral	3 (6)	6 (12)	7 (14)		
	Disagree	2 (4)	1 (2)	2 (4)		
	Strongly disagree	2 (4)	1 (2)	2 (4)		
Degree of interest in information technology devices (1 to 5), mean (SD)		3.84 (1.10)	4.06 (0.87)	3.88 (0.90)	.48	
Frequency of use of information technology devices (1 to 5), mean (SD)		4.02 (1.04)	4.12 (0.90)	3.92 (0.97)	.59	

^a^ORT: oscillating-rotating power toothbrush.

^b^ITT: interactive telemonitoring toothbrush.

^c^MT: manual toothbrush.

^d^1-way ANOVA for continuous variables and chi-square test categorical variables.

### Changes in SHS, QHI, and H2S and CH3SH Levels

**Figure 3:**
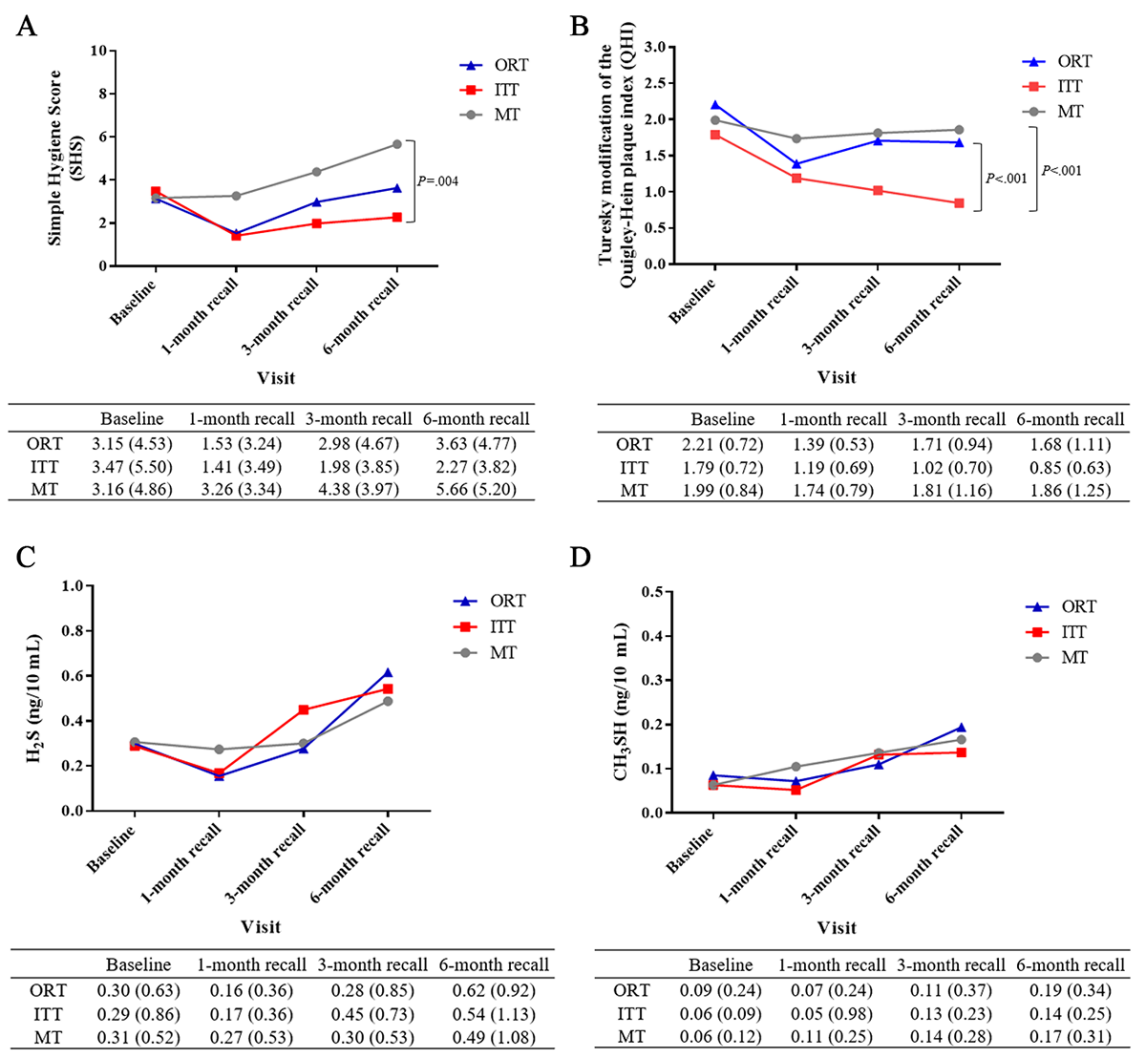
Comparison of the mean (SD) of the (A) Simple Hygiene Score (SHS), (B) Turesky modification of the Quigley-Hein plaque index (QHI), and halitosis in terms of (C) H2S and (D) CH3SH values between the oscillating-rotating power toothbrush (ORT), interactive telemonitoring toothbrush (ITT), and manual toothbrush (MT) groups uing 2-way, repeated-measures ANOVA with the Bonferroni multiple comparisons test.

The mean SHS increased over time in the MT group (3.16, SD 4.86 to 5.66, SD 5.20) and decreased at the 1-month recall in the ORT and ITT groups (1.53, SD 3.24 and 1.41, SD 3.49, respectively). Compared with the MT group, the SHS significantly decreased over time in the ITT group (*P*=.004; Figure 3A). Regarding QHI, the mean scores decreased from baseline at the 1-month recall in all 3 groups (ORT group: 2.2, SD 0.72 to 1.39, SD 0.53; ITT group: 1.79, SD 0.72 to 1.19, SD 0.69; MT group: 1.99, SD 0.84 to 1.74, SD 0.79). However, at the 6-month recall, the scores in the ORT and MT groups increased (ORT group: 1.68, SD 1.11; MT group: 1.86, SD 1.25), while that in the ITT group decreased (0.85, SD 0.63; *P*<.001; Figure 3B). However, there were no significant differences in H_2_S and CH_3_SH levels among the 3 groups during the entire study period (Figures 3C and 3D).

### Microbiota Changes

**Figure 4:**
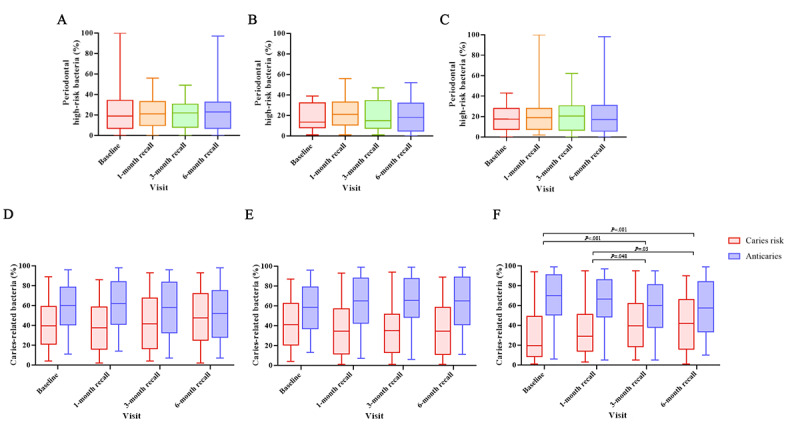
At each of the study time points, boxplots of the percentages of periodontal high-risk, caries-related microbiota in the (A) oscillating-rotating power toothbrush (ORT), (B) interactive telemonitoring toothbrush (ITT), and (C) manual toothbrush (MT) groups, with the boxes representing the 25th to 75th percentiles and the line representing the median, as well as the ratio of caries-risk to anticaries microbiota in the (D) ORT, (E) ITT, and (F) MT groups at the different time points, with changes in the ratios over time analyzed using paired-samples t tests.

Regarding the microbiota, there were no significant differences in the high-risk periodontal microbiota over time among the 3 groups (Figures 4A-4C). Likewise, there was no significant difference in the ratio of caries-risk microbiota to anticaries microbiota between the ORT and ITT groups (Figures 4D and 4E). However, in the MT group, the ratio of caries-risk microbiota significantly increased at the 3-month (*P*<.001) and 6-month (*P*=.005) recalls compared with baseline and at the 3-month (*P*=.048) and 6-month (*P*=.03) recalls compared with the 1-month recall. Conversely, the ratio of anticaries microbiota significantly decreased at the 3-month and 6-month recalls (Figure 4F).

### Self-Reported Questionnaire and Toothbrushing Behavior Changes

The groups completed a self-reported questionnaire on current oral care habits and awareness of telemonitoring toothbrushes after finishing the study. On the questionnaire, the most common answers to the question regarding the most effective function of a telemonitoring toothbrush were the brushing guide function by the ORT and MT groups and the standard brushing training by the ITT group (*P*<.001; Table 2). The most inconvenient feature of the telemonitoring toothbrush was inaccurate toothbrush position recognition, according to participants in the ORT and ITT groups (*P*<.001). In the ORT and ITT groups, 88% (44/50) and 82% (41/50) of the participants, respectively, indicated that the smart toothbrush was helpful for oral health, and 76% (38/50) and 92% (46/50) of the participants, respectively, reported that their brushing ability had improved. Mean satisfaction with the smart toothbrush was 7.90 (SD 1.21) and 7.11 (SD 1.67) in the ORT and ITT groups, respectively; these were significantly lower in the ITT group than in the ORT group (*P*=.008). The need for a smart toothbrush was not significantly different between the ORT (mean 7.44, SD 1.64) and ITT (mean 7.71, SD 1.67) groups.

**Table 2:** Postquestionnaire responses on oral care habits and awareness of telemonitoring toothbrushes.

Questions			ORT^a^ (n=50)		ITT^b^ (n=50)		MT^c^ (n=50)		*P* value^d^	
Number of toothbrushing events per day, mean (SD)			2.36 (0.60)		2.60 (0.61)		2.62 (0.57)		.053	
Length of teethbrushing per toothbrushing event (minutes), mean (SD)			3.04 (0.49)		3.28 (0.67)		3.46 (0.84)		.01	
**What are the most valid functions of a telemonitoring toothbrush?, n (%)**										<.001
	Function of guiding brushing	22 (44)		17 (34)		27 (55)				
	Management of brushing history	20 (40)		6 (12)		9 (18)				
	Standard brushing training	7 (14)		25 (50)		12 (25)				
	Other	1 (2)		2 (4)		1 (2)				
**What is the most inconvenient thing about a telemonitoring toothbrush?, n (%)**										<.001
	Adjusting smartphone position	9 (18)		8 (16)		24 (48)				
	Inaccurate toothbrush position recognition	39 (78)		34 (68)		16 (32)				
	Signing up	0		1 (2)		5 (10)				
	Management of brushing history	0		0		4 (8)				
	Other	2 (4)		7 (14)		1 (2)				
**Do you think a telemonitoring toothbrush can benefit your oral health?, n (%)**										.66
	Strongly agree	10 (20)		6 (12)		6 (12)				
	Agree	34 (68)		35 (70)		32 (64)				
	Neutral	6 (12)		8 (16)		11 (22)				
	Disagree	0		1 (2)		1 (2)				
	Strongly disagree	0		0		0				
**Do you think your brushing ability is better than before?, n (%)**										.12
	Strongly agree	11 (22)		9 (18)		—^e^				
	Agree	27 (54)		37 (74)		—				
	Neutral	9 (18)		3 (6)		—				
	Disagree	3 (6)		1 (2)		—				
	Strongly disagree	0		0		—				
**Are you willing to buy a telemonitoring toothbrush and use it continuously?, n (%)**										.002
	Strongly agree	14 (28)		3 (6)		2 (4)				
	Agree	21 (42)		17 (34)		24 (48)				
	Neutral	8 (16)		24 (48)		18 (36)				
	Disagree	6 (12)		4 (8)		5 (10)				
	Strongly disagree	1 (2)		2 (4)		1 (2)				
**If you could receive oral care services at a general dentist as a result of using a telemonitoring toothbrush, would you visit that dentist?, n (%)**										.17
	Strongly agree	6 (12)		6 (12)		7 (14)				
	Agree	24 (48)		33 (66)		36 (72)				
	Neutral	15 (30)		10 (20)		5 (10)				
	Disagree	4 (8)		1 (2)		2 (4)				
	Strongly disagree	1 (2.0)		0		0				
Satisfaction with the telemonitoring toothbrush (1 to 10), mean (SD)			7.90 (1.21)		7.11 (1.67)		0		.008	
Need for a telemonitoring toothbrush (1 to 10), mean (SD)			7.44 (1.64)		7.71 (1.67)		0		.42	

^a^ORT: oscillating-rotating power toothbrush.

^b^ITT: interactive telemonitoring toothbrush.

^c^MT: manual toothbrush.

^d^1-way ANOVA for continuous variables and chi-square test categorical variables.

^e^Not applicable.

When comparing the average number of toothbrushing events and minutes per toothbrushing event by group, at baseline and after study completion, the number of toothbrushing events decreased significantly in the ORT group (*P*=.02) and slightly in the ITT and MT groups (Table 3). The average number of minutes spent per toothbrushing event increased in all 3 groups, especially in the MT group (*P*=.01).

**Table 3:** Comparison of changes in brushing habits by group.

Variable		Before (baseline)	After (6-month recall)	*P* value^a^
**Number of toothbrushing events, mean (SD)**				
	ORT^b^ (n=50)	2.54 (0.61)	2.36 (0.60)	.02
	ITT^c^ (n=50)	2.72 (0.61)	2.60 (0.61)	.057
	MT^d^ (n=50)	2.70 (0.61)	2.62 (0.57)	.32
**Length of each toothbrushing event (minutes), mean (SD)**				
	ORT (n=50)	2.86 (0.76)	3.04 (0.49)	.12
	ITT (n=50)	3.10 (0.81)	3.28 (0.67)	.06
	MT (n=50)	3.12 (0.82)	3.46 (0.84)	.01

^a^*t* test.

^b^ORT: oscillating-rotating power toothbrush.

^c^ITT: interactive telemonitoring toothbrush.

^d^MT: manual toothbrush.

## Discussion

### Principal Findings

This study demonstrated that ITTs could improve oral hygiene management. The SHS and QHI, indicators of oral hygiene, improved significantly more for the ITT group than the ORT and MT groups when the values at baseline and the 6-month recall were compared between the groups. Along with these results, changes in the oral microbiota were also confirmed. We classified *Streptococcus sanguinis* as anticaries microbiota and compared its quantitative ratio against caries-risk microbiota, such as *Streptococcus mutans, Streptococcus sobrinus, Actinomyces gerencseriae, Scardovia wiggsiae, Veillonella parvula*, and *Candida albicans*. In the ITT group, the proportion of anticaries microbiota increased over time. In the ORT group, the proportion of anticaries microbiota increased until the 3-month recall; however, it decreased at the 6-month recall. In the MT group, the proportion of anticaries microbiota continued to decrease at all time points. Although reducing caries-risk microbiota is important, increasing anticaries microbiota is also an important part of oral hygiene [17,18]. These findings highlight the importance of compliance and sustained engagement in oral hygiene maintenance, which was facilitated by continuous feedback in the ITT group.

### Comparison With Prior Work

The importance of compliance effects for smartphone-based digital health care devices has been reported in previous studies. For example, a telemonitoring application for blood glucose management by individuals with diabetes was effective at the beginning of use, but as satisfaction with the application decreased, the effectiveness also decreased [19]. Using telemonitoring to control asthma has also been reported to be effective. However, a higher level of outpatient care was received compared with national averages [20]; therefore, we cannot rule out the possibility that this may have had an impact on compliance with continued application use.

Based on the questionnaire administered before and after the study, 83% of the participants in the ORT and ITT groups who used smart toothbrushes responded that their brushing abilities improved. Among the 2 groups using smart toothbrushes, the SHS and QHI improved more in the ITT group, whereas satisfaction with the smart toothbrush was higher in the ORT group. This might be due to Oral-B’s fancy hardware and application appearance and easy-to-use charging method compared with the ITT.

In the 2000s, an interactive toothbrush equipped with a monitoring function using toothbrushing and grip axis recognition was introduced [21]. With the advancement in modern technology, it has become possible to provide instructional brushing videos on smartphones and app-based brushing monitoring using real-time motion recognition via Bluetooth [22]. In addition, messages and chat apps are known to help improve brushing, and recently released smart toothbrushes are equipped with various functions, such as messages and motion recognition. An interactive toothbrush with a smartphone app has the advantage of recording toothbrushing data, enabling dental specialists to provide personalized feedback and improve habits through toothbrushing notifications [23,24]. In particular, these findings suggest that children and adolescents who use an interactive toothbrush will benefit from plaque removal and improved gingival health [25-27]. We believe that our study is meaningful in that we have suggested an optimal oral hygiene management method for patients, by comparing clinical efficacy not only between interactive and manual toothbrushes but also between 2 different smart toothbrushes.

The medical paradigm has recently changed to include quick information use and interactive communication using smartphones, which are gradually being used for telemedicine, remote monitoring, and health intervention provision [28-30]. In dentistry, apps to improve oral hygiene are used in various ways, and many studies have verified their effectiveness [31,32]. These innovative smartphone-based mobile apps are also being used as digital therapeutics. Digital therapeutics and software medical devices that provide evidence-based therapeutic interventions to prevent, manage, and treat medical disorders or diseases have been launched in various countries [33]. Moreover, the results of this study showed that smart toothbrushing is not limited to hardware only. An interactive app that encourages the correction of toothbrushing habits has the potential to be used as a digital treatment. In addition, although the duration of and scores for toothbrushing temporarily improved immediately after recall in this study, it was confirmed that interest in brushing had decreased at the 3-month and 6-month recalls, which was probably a long recall period. This suggests that, when using a smart toothbrush, the recall interval with a dental professional should not be too long to promote effective teledentistry treatments, such as a digital therapeutic, and that patient compliance with the smart toothbrush is important.

### Strengths and Limitations

#### Strengths

This study is one of few to comprehensively compare an ITT and ORT with an MT, evaluating their effectiveness in clinical, microbiological, and user experience dimensions. A robust randomized controlled trial design with clearly defined interventions and outcomes ensured the reliability of the findings.

#### Limitations

The limitations of this study are as follows. First, we restricted the use of auxiliary oral hygiene devices, such as dental floss and interdental brushes, during the study period. Although the use of auxiliary oral hygiene devices was restricted to compare the efficacy of the smart toothbrush itself, the use of auxiliary oral hygiene devices plays an important role in oral hygiene management in interdental areas [34]. Since auxiliary oral hygiene devices are highly recommended in clinical practice, it is necessary to evaluate plaque removal and gingival health improvement when using auxiliary oral hygiene devices together with a smart toothbrush. Second, the nutrition practices of the participants were not recorded. Nutrition practices could affect the oral environment in addition to brushing, but this was not considered in the result analysis. Third, right-handedness and left-handedness were not considered. Since the app showed a video guide on how to brush based on right-handedness, it could have been confusing for left-handed participants. Finally, participants were recruited from specific higher education institutions, resulting in a younger demographic. Additionally, as participants responded to a recruitment posting, they may have been more motivated or interested in oral health than the general population, potentially limiting the generalizability of the findings.

### Conclusions

The ITT group had significant improvements on the SHS and QHI, demonstrating that telemonitoring toothbrushes enable proper oral hygiene management compared with manual and oscillating-rotating toothbrushes. This highlights the importance of expert feedback through the transmission of users’ toothbrushing data. However, it was confirmed that interest in toothbrushing declined at the 3-month and 6-month recalls, which were long recall periods. In the future, additional research will be needed on the appropriate feedback cycle and compliance with the app by participants.

## Appendix

Multimedia Appendix 1 CONSORT-eHEALTH checklist (V 1.6.1).

Multimedia Appendix 2 Example of Mombrush ProCare application with Bluetooth connection to Mombrush, an interactive telemonitoring toothbrush.

Multimedia Appendix 3 Example of the Oral-B mobile application connecting via Bluetooth to the Oral-B Genius 8000 oscillating-rotating electric toothbrush.

## Abbreviations

CONSORT-EHEALTH: Consolidated Standards of Reporting Trials of Electronic and Mobile Health Applications and Online Telehealth
IRB: Institutional Review Board
ITT: interactive telemonitoring toothbrush
MT: manual toothbrush
ORT: oscillating-rotating power toothbrush
PCR: polymerase chain reaction
QHI: Turesky modification of the Quigley-Hein plaque index
SHS: Simple Hygiene Score

## Data Availability

Data that support the findings of this study are available from the corresponding authors upon request and following institutional review board rules and privacy regulations.
