# Supplementary material for: Smartphone-Based Telemonitoring for Better Oral Health With Toothbrushes: 6-Month Randomized Controlled Trial
Source: J Med Internet Res. 2025 Feb 10;27:e65128. doi: 10.2196/65128 (PMC11851029; doi:10.2196/65128)

Multimedia Appendix 1: Example of Mombrush ProCare application with Bluetooth connection to Mombrush®, an interactive telemonitoring toothbrush

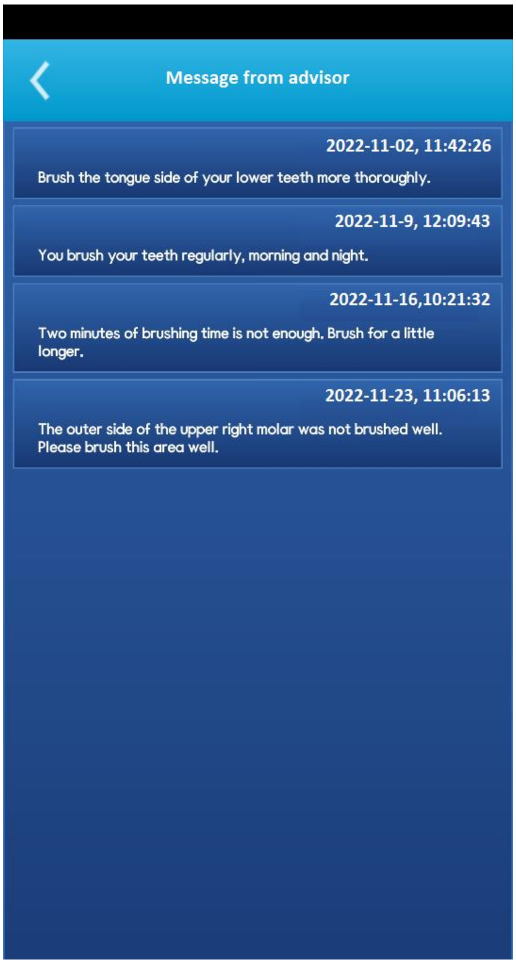

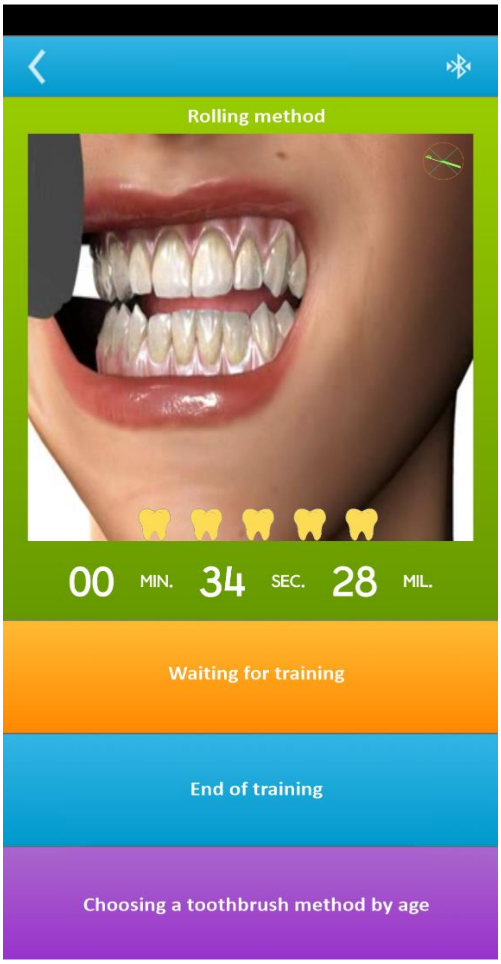

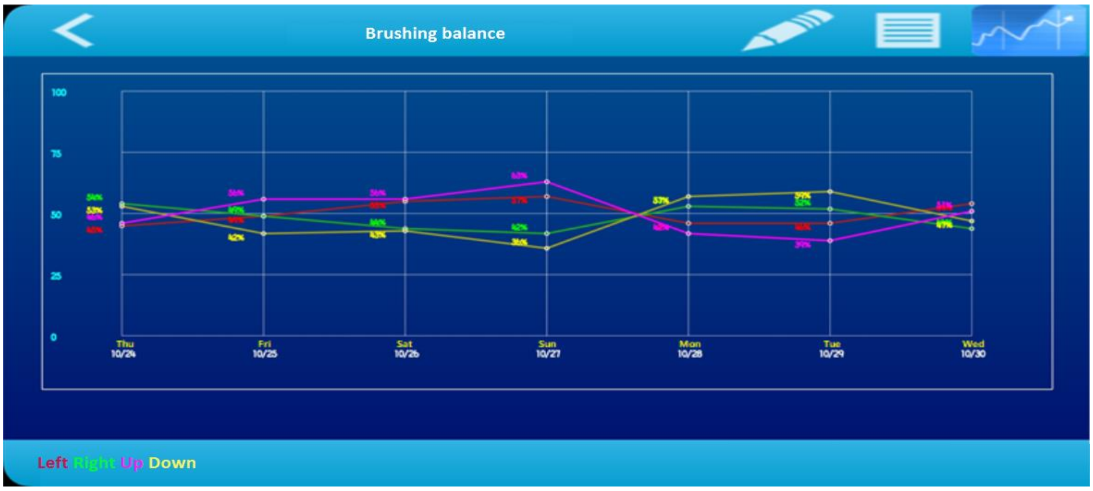

Supplement: Multimedia Appendix 2 [file jmir_v27i1e65128_app2.pdf]
