# Supplementary material for: Smartphone-Based Telemonitoring for Better Oral Health With Toothbrushes: 6-Month Randomized Controlled Trial
Source: J Med Internet Res. 2025 Feb 10;27:e65128. doi: 10.2196/65128 (PMC11851029; doi:10.2196/65128)

Multimedia Appendix 2: Example of the Oral-B mobile application connecting via bluetooth to the Oral-B® Genius 8000 oscillating-rotating electric toothbrush

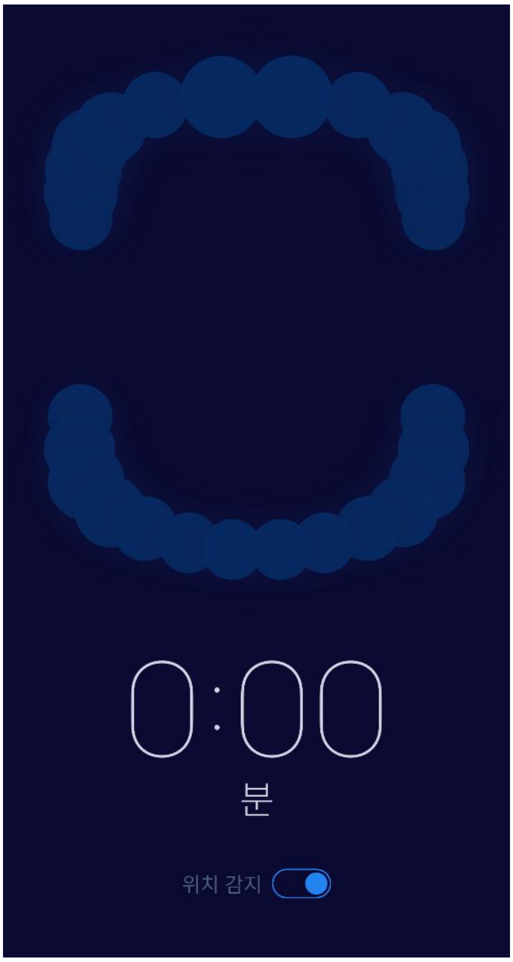

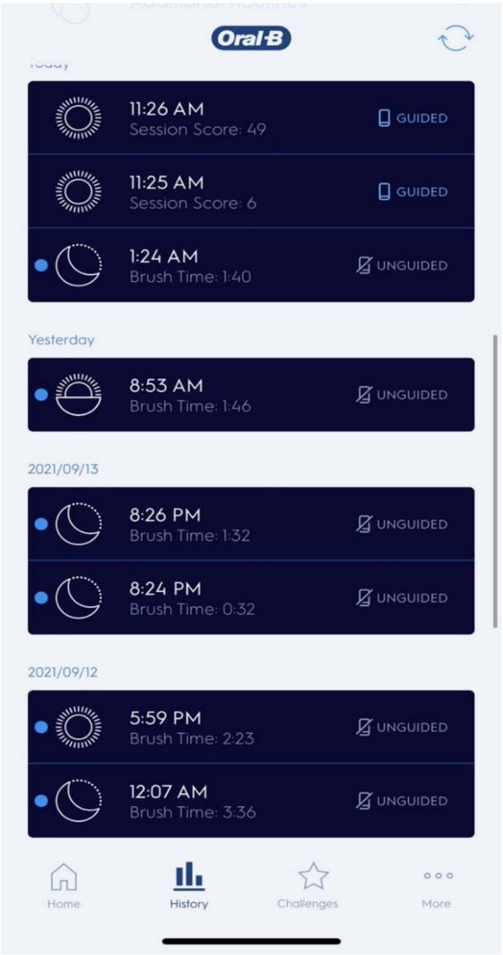

Supplement: Multimedia Appendix 3 [file jmir_v27i1e65128_app3.pdf]
